# Approaching Genetics Through the MHC Lens: Tools and Methods for HLA Research

**DOI:** 10.3389/fgene.2021.774916

**Published:** 2021-12-02

**Authors:** Venceslas Douillard, Erick C. Castelli, Steven J. Mack, Jill A. Hollenbach, Pierre-Antoine Gourraud, Nicolas Vince, Sophie Limou

**Affiliations:** ^1^ Centre de Recherche en Transplantation et Immunologie, CHU Nantes, Inserm, Centre de Recherche en Transplantation et Immunologie, Université de Nantes, Nantes, France; ^2^ Unesp—Universidade Estadual Paulista, Botucatu, Brazil; ^3^ Division of Allergy, Immunology and Bone Marrow Transplantation, Department of Pediatrics, School of Medicine, University of California, San Francisco, San Francisco, CA, United States; ^4^ Department of Neurology, University of California, San Francisco, San Francisco, CA, United States; ^5^ Department of Epidemiology and Biostatistics, University of California, San Francisco, San Francisco, CA, United States; ^6^ Ecole Centrale de Nantes, Department of Computer Sciences and Mathematics in Biology, Nantes, France

**Keywords:** Major Histocompatibility Complex (MHC), HLA, association analysis, imputation, immunogenetics

## Abstract

The current SARS-CoV-2 pandemic era launched an immediate and broad response of the research community with studies both about the virus and host genetics. Research in genetics investigated HLA association with COVID-19 based on *in silico*, population, and individual data. However, they were conducted with variable scale and success; convincing results were mostly obtained with broader whole-genome association studies. Here, we propose a technical review of HLA analysis, including basic HLA knowledge as well as available tools and advice. We notably describe recent algorithms to infer and call HLA genotypes from GWAS SNPs and NGS data, respectively, which opens the possibility to investigate HLA from large datasets without a specific initial focus on this region. We thus hope this overview will empower geneticists who were unfamiliar with HLA to run MHC-focused analyses following the footsteps of the Covid-19|HLA & Immunogenetics Consortium.

## Introduction to Human Leukocyte Antigens: Creating Immunity From Diversity

The classical HLA proteins are expressed on the surface of human cells. Although their primary role is to present exogenous and endogenous peptides, they were first described as “antigens” due to their interaction with T-cells in transplant rejection (Dausset, 1958). Along with other genes in the *MHC* region, the products of the *HLA* genes are essential in the adaptive immune response. By presenting peptides to both CD8^+^ (HLA class I molecules) and CD4^+^ T cells (HLA class II molecules), HLA proteins initiate an immune response against foreign (non-self) peptides which may be defective products of translation, neo-antigens generated by mutated genes from tumor cells, or pathogenic in origin. In addition, class I HLA proteins interact with the KIR ligands of NK cells, including KIR and LILRB, which are important in innate immunity (Carrington et al., 2008; Kulkarni et al., 2008; Trowsdale and Moffett, 2008). Thus, HLA molecules are key features of both innate and adaptive immune responses. *HLA* genes central role in immunity against infectious diseases and their importance for transplantation have made them the subject of much study.

HLA proteins are coded by multiple genes on the short arm of chromosome 6 at the 6p21 locus; this region containing *HLA* genes is referred to as the Major Histocompatibility Complex (MHC) for its seminal role in transplantation (Dausset, 1981; Montgomery et al., 2018). Although there is a common confusion between the two terms *HLA* and *MHC*, *HLA* specifically refers to the genes involved in antigen processing and presentation whereas the *MHC* corresponds to a whole locus, with *HLA* and other immune-related genes such as the complement system. The *MHC* region is the most gene-dense region of the human genome, with 1% of the human coding genes (>200) found in 0.1% of the genome length (Shiina et al., 2009). The *MHC* region is commonly defined as a 4 Mb segment on chromosome 6 (*MOG* 29657002–33192499 *COL11A2*, GRCh38. p13 assembly) (Beck et al., 1999). However, due to extended patterns of linkage disequilibrium (LD), an extended MHC (xMHC) is often referred to in immunogenomics (25726063–33400556, GRCh38. p13 assembly) (Horton et al., 2004). The *MHC* region is divided into three regions based on gene sequence similarities and functions, class I, II, and III in which approximately 40% of the genes are immune-related. *HLA* genes are found in the class I and class II regions and are commonly divided in two categories: classical HLA proteins present peptides to T-cells, whereas non-classical HLA are mostly involved either in peptide presentation with other receptors, with immune modulation, or with various steps of classical HLA formation and loading.

The *MHC* class I region contains 12 *HLA* pseudogenes and 6 *HLA* genes (*HLA-A*, -*B*, -*C*, -*E*, -*F,* and -*G*), including three classical (*HLA-A*, -*B,* and -*C*) that are ubiquitously expressed as a heterodimer with beta-2 microglobulin at the cells’ surface. Class I HLA molecules and their bound peptides are specifically recognized by CD8^+^ T cells receptors. The non-classical HLA class I molecules (*HLA-G*, *-E*, and *-F*) present different expression patterns. *HLA-E* and *HLA-F* are usually ubiquitously expressed in low levels, and they interact with ligands in T and NK cells (such as HLA-E with NKG2A). *HLA-G* is predominantly expressed at the maternal-fetal interface and has primarily been associated with maternal–fetal tolerance by interacting CD8 from T cells and LILRB1, LILRB2, and KIR2DL4 from NK cells (Donadi et al., 2011).

The Class II region comprises four non-classical genes (*HLA-DMA*, -*DMB*, -*DOA*, -*DOB*), mostly related to peptide loading, and 17 classical *HLA* genes (e.g., *HLA-DRA*, *HLA-DRB1*, *HLA-DQA1*, *HLA-DQB1*, *HLA-DPA1*, and others) that are expressed in Antigen Presenting Cells (APC) such as B cells, monocytes, macrophages, dendritic cells as well as epithelial cells under inflammatory signals (Rock et al., 2016). Unlike class I HLA molecules, class II molecules are heterodimers, consisting of α and β chains, encoded by the corresponding *HLA* genes (e.g., *HLA-DPA1* and *HLA-DPB1* produce the HLA-DP molecule), which facilitates molecular diversity. The HLA-DR beta chain can be encoded by nine different genes (DRB1-9) with complex patterns of expression, and gene content adding additional layers of complexity (Faner et al., 2009). Finally, the class III region, located between the class I and II regions, is the most gene-dense region of the *MHC*; this region contains genes encoding elements of the complement system, chaperone genes, cytokines such as *TNF* and *LTA*, but no *HLA* genes.

Finally, there are other important non-*HLA* genes in the *MHC*, such as *TAP1* and *TAP2*, both related to peptide pumping from the cytoplasm to the endoplasmic reticulum (Praest et al., 2018), *MICA* and *MICB*, both induced in viral infections and tumors and activate NK-mediate killing (Ghadially et al., 2017), the tripartite motif (TRIM) family, related to cell cycle progression, autophagy, and viral replication restriction (van Tol et al., 2017), *PSORS1C1*, conferring susceptibility to psoriasis and systemic sclerosis (Allanore et al., 2011), and others.

In addition to their large number and potential for many combinations, the *HLA* genes display unparalleled genetic diversity, with more than 27,000 alleles and almost 17,000 unique proteins (June 03, 2021, https://www.ebi.ac.uk/ipd/imgt/hla/stats.html) identified for the five most polymorphic loci (*HLA-A*, *-C*, *-B*, *-DRB* and *-DQB1*). This diversity of HLA molecules is concentrated in the peptide-binding groove, which allows the presentation of peptides of various shapes and sizes, hence conferring broad protection against pathogens at the population level. At the same time, the polymorphic nature of *HLA* is also found on non-coding parts of the genes, such as the promoter and have an impact on expression (Kulkarni et al., 2011; Vince et al., 2016; Lima et al., 2019). Over evolutive time, together with founder effects, multiple pathogen-challenges have exerted selective pressures on *HLA* alleles in human populations across the globe (Meyer and Thomson, 2001; Spurgin and Richardson, 2010), shaping allele frequency differences and selecting very specific or even private *HLA* alleles in some populations (Brandt et al., 2018; Meyer et al., 2018). The progress of genomics, and immunogenomics over the last decade, had deepened our understanding of HLA role in human diseases though the use of genome-wide association studies (GWAS) (Kennedy et al., 2017; Dendrou et al., 2018).

The COVID-19 HLA and Immunogenetics Consortium (CHIC) has been created during the pandemic to coordinate efforts on *HLA* analysis. The CHIC provided a website with information on HLA data and current projects (The COVID-19 HLA and Immunogenetics Consortium, 2020a). It is supported by a database (The COVID-19 HLA and Immunogenetics Consortium, 2020b) and its role is the centralization of relevant HLA and clinical data for COVID-19 study. It contains HLA data of 2,892 individuals from nine projects. These data are freely available and new data can be easily uploaded upon account creation. In addition, the website allows HLA allele frequencies visualization, and use of HLA data management and analysis tools. An HLA Imputation Portal (HIP) is set up to allow geneticists to infer individuals HLA alleles using SNP genotyping data, relying on multi-ethnic models from Zheng et al. (Zheng et al., 2014). This tool may help leverage SNP data to gain power in HLA association studies. The CHIC also produced a broad review on immunogenetic parameters (e.g., HLA, KIR, complement, cytokines and chemokines receptors) and their role in COVID-19 (Aguiar et al., 2021). A more specific review of COVID-19 and HLA associations (Douillard et al., 2021) highlights links between the pathology and HLA at different levels, from allele frequency correlation to HLA associations and haplotypes. The consortium will gradually improve its portal by providing access to additional and more diverse imputation reference panels, and by recruiting more individuals. Results from GWASs showed no association between HLA SNPs and COVID-19 infection (COVID-19 Host Genetics Initiative, 2021) but demonstrated an association with COVID-19 severity; dedicated HLA allele association studies identified potential signals of interest (Castelli et al., 2021). The spread of HLA tools, allowing HLA allele inference from whole-exome or whole-genome sequencing as well as from GWAS SNP data will significantly increase the sample size from available cohorts to maximize the statistical discovery power of HLA-centric studies. In this report, we pursue this effort to provide an overview of methods for generating HLA data along with several analytical strategies to capitalize on this genetic information. We will also cover additional immunogenomic parameters, as MHC-related associations still have much to reveal (Trowsdale and Knight, 2013). We hope this work will empower researchers to include HLA-focused investigations in their palette and will contribute to promote efforts for in-depth explorations of the relationship between HLA and immune-related outcomes in this pandemic era.

## Generating and Working With HLA Data

Performing immunogenetic studies can be a challenge for those unfamiliar with the specifics of HLA nomenclature. An individual HLA genotype can be obtained through multiple molecular techniques, the complexity of its nomenclature allows the alleles in a genotype to be described in different styles, and these data can be stored in a variety of file standards. Overall, HLA information can take multiple forms, requiring a comprehensive understanding of the nomenclature in order to run proper statistical analyses and find relevant associations.

### Generating HLA Data

Originally, immunologists conducted microlymphocytotoxicity assays, testing patients T/B cells (for HLA class I) or B cells (for HLA class II) against different anti-sera or monoclonal antibodies in the presence of complement. Sera or antibodies recognizing the HLA antigens on cells would activate the complement and lyse the cell; this serology staining would reveal the patient HLA serotype (Park and Terasaki, 2000). Serology was however limited by the underlying complexity of HLA and it resulted in poor performances in transplantation (Hurley, 2021). The need to improve this performance and technique evolution, with the advent of PCR, conducted HLA specialists to switch to molecular typing. Molecular techniques were adopted for *HLA* typing; these methods allowed systematic identification of *HLA* alleles, based on sequence polymorphisms, providing a ‘higher resolution’ result that distinguishes many more allele categories than serological methods. This molecular typing consistently improved in resolution throughout the years driving nomenclature evolution along the way. Sequence-specific (PCR-SSO) methods rely on the hybridization of hundreds of labeled SSO probes targeting unique sequences in polymorphic regions. Sequence-specific priming (PCR-SSP) methods directly amplify elements of the *HLA* genes with PCR primers containing sequence-specific 3′ end polymorphisms, resulting in less ambiguity (inability to distinguish alleles with similar nucleotide sequences), than SSO methods (Meral and Beksaç, 2007).

Sanger sequencing-based typing (PCR-SBT) methods initially provided sequences of the exons that encoded the peptide-binding groove, and later overlapping sets of sequences for entire genes. PCR-SBT was the gold standard for *HLA* genotyping until the development of next-generation sequencing (NGS) methods (Meral and Beksaç, 2007; De Santis et al., 2013). Except for the last one, PCR-SBT, previous methodologies were not suitable to detect new variants, and their goals were detecting known polymorphisms.

The application of NGS was explored in 2012, as part of the 16th International HLA and Immunogenetics Workshop (IHIW), but, given issues with mapping of short reads, allelic imbalance, phasing, and high costs, PCR-SBT remained the gold standard. More recently, the integration of NGS technologies with bioinformatic solutions for immunogenetics has improved the speed and accuracy of NGS *HLA* genotyping with lower error rates and fewer ambiguities than PCR-SBT (Baier et al., 2019; Jekarl et al., 2021), and the application of NGS was the focus of the 17th IHIW in 2017 (Vayntrub et al., 2020). Moreover, NGS is ideal to detect new *HLA* variants. Researchers now routinely identify novel *HLA* alleles (Nilsson et al., 2018; Ralazamahaleo et al., 2019; Loginova et al., 2020; Ananeva et al., 2021a; Ananeva et al., 2021b; Cheranev et al., 2021; Loginova et al., 2021) using NGS and confirm them using SBT with PCR-SBT error often responsible for non-concordance between the two. NGS-based sequencing of multiple exons and introns has led to increases in the growth of the IPD-IMGT-HLA Database collection (Robinson et al., 2015; Robinson et al., 2019). Unfortunately, the total number of new alleles may be underestimated as it is not uncommon for new alleles to be NGS-typed without Sanger validation.

So-called third-generation NGS generates unambiguous, phased *HLA* genotypes, using instruments like the PacBio SMRT (Mayor et al., 2015) or Oxford Nanopore Technology MinION (De Santis et al., 2020) to avoid multiple molecular techniques. This approach is faster than SBT and generates phased polymorphism with longer reads. Researchers using Oxford Nanopore Technology systems have successfully sequenced 11 *HLA* loci with low ambiguities in under 6 h (Mosbruger et al., 2020).

### *HLA* Nomenclature

Soon after cell-surface antigens were identified as polymorphic between individuals, the WHO Nomenclature Committee for Factors of the HLA System was formed to develop a specific nomenclature for *HLA* genes, proteins and allelic variants (Allen et al., 1968). The original “HL-A″ factor serologically typed with multiple antibodies with an individual type (e.g., HL-A (1,2/7,8) identifying them as positive for factors 1,2,7,8, and confirmed two distinct haplotypes from parental typing. As dozens of *HLA* genes and thousands of alleles were identified, the nomenclature was expanded to accommodate new complexity while building on the historical serological vocabulary. In 1987, the nomenclature was updated to accommodate newly available protein and nucleotide sequences (Antigens, 1987). The modern locus names were adopted at this time, and four-digits names were assigned to alleles, which were only defines as protein variants. In 2010, the current field-delimited nomenclature was adopted to account for the growing number of silent and non-coding nucleotide variants (Marsh et al., 2010).

**FIGURE 1:**
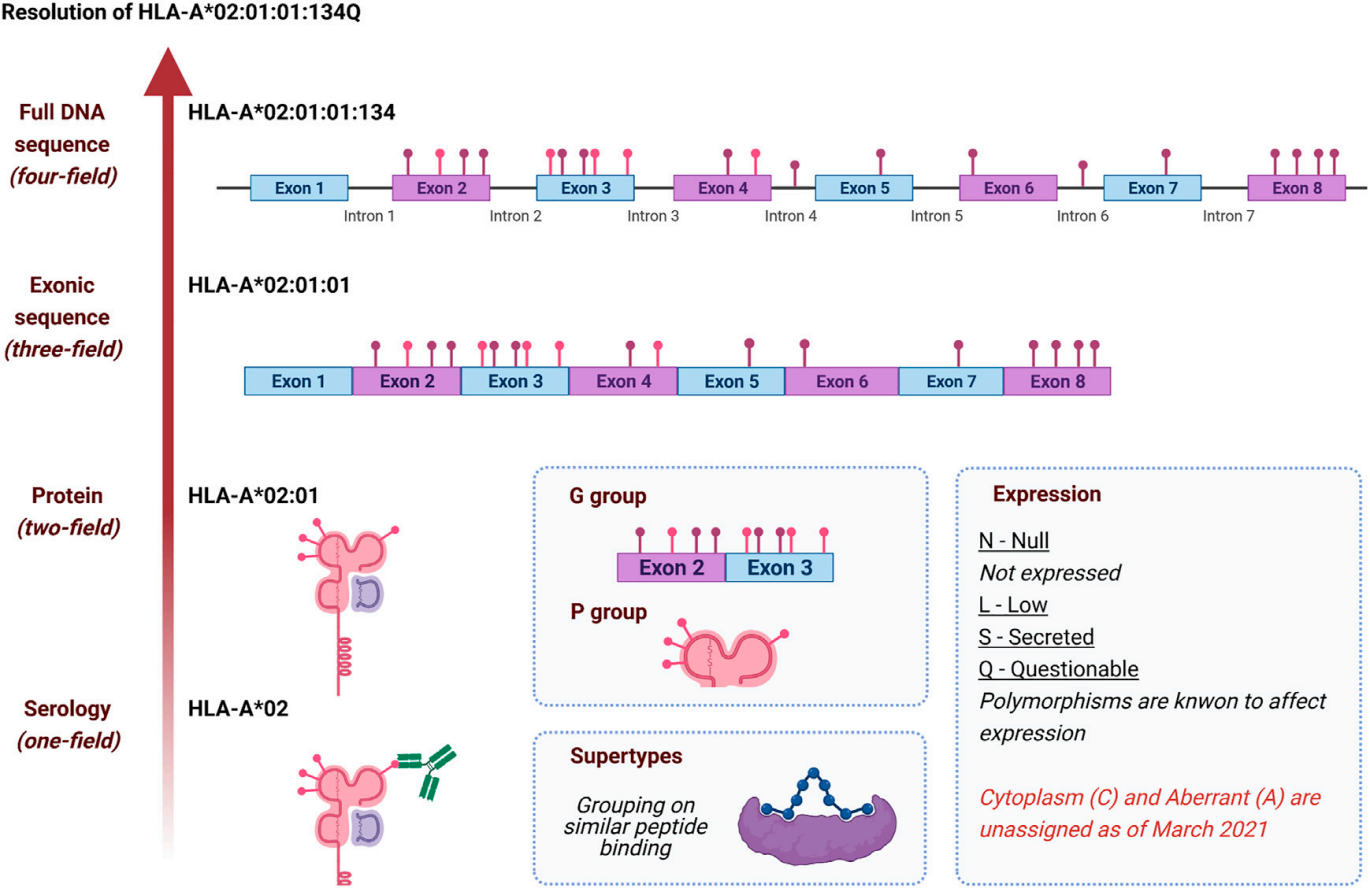
History and development of HLA nomenclature as illustrated by HLA-A*02:01:01:134Q. Each level of resolution corresponds to a group of *HLA* alleles fitting the description, except for the full DNA sequence, a unique *HLA* allele. Colored pins represent non-synonymous polymorphism (pink) and synonymous or intronic polymorphisms (purple); the displayed polymorphism is only indicative and does not reflect HLA-A*02:01:01:134Q sequence. P and G groups are named with the lowest numbered two-field (HLA-A*02:01) and three-field (HLA-A*02:01:01) HLA allele name, respectively. Class II P and G groups are based on exon 2 only, while class I P and G groups are based on exons 2 and 3. Supertypes are not defined as part of the official nomenclature (Wang and Claesson, 2014; del Guercio et al., 1995; Sidney et al., 1995). Created with biorender.com.

A modern HLA allele name consists of up to four “fields”, each of which includes a two- or more digit number, each separated by a colon (Figure 1).

The first and second fields represent a historical serological group, and a unique protein sequence, respectively. All allele names have at least two fields. Alleles sharing the 1st, and 2nd fields with a different 3rd field encode the same protein but have unique silent-substitution in the exonic sequence, whereas sequence differences contained in the introns are written in the 4th field. The four fields of an allele name can also be suffixed with a single-letter “expression variant”, identifying alleles that are either not expressed, expressed at a low or questionable level, or secreted. For example, HLA-A*02:01:01 represents an exonic sequence shared by e.g., HLA-A*02:01:01:01 and HLA-A*02:01:01:134Q. In the latter case, the expression of HLA-A*02:01:01:134Q is Questionable, due to a potential alternate splicing nucleotide variant in intron 2. Allele names can be truncated to fewer fields for different applications, with each truncation described as a level of “resolution” (e.g., HLA-A*02 is a one-field resolution allele).

In addition to this allele nomenclature, specific groups of alleles have been defined. P and G groups refer to multiple alleles sharing either the same peptide or nucleotide sequence for the peptide-binding groove, respectively. For instance, HLA-A*02:01:01:134Q and HLA-A*02:252 both belong to the A*02:01P P group; the two proteins are globally different but share the same peptide-binding groove. HLA-A*02:01:01:134Q and HLA-A*02:89:01 belong to the A*02:01:01G G group as they share identical peptide-binding groove encoding exon sequences.

HLA supertypes are groups of alleles sharing similar peptide-binding repertoires. Supertypes are defined by “structural similarities, shared peptide-binding motifs, and identification of cross-reacting peptides” (Wang and Claesson, 2014). Using this classification, HLA-A*02:01:01:134Q potentially belongs with HLA-A*02:02, A*02:05*,* A*69:01 in the A2 supertype. (del Guercio et al., 1995). Some studies of the HLA molecules’ evolution have interpreted HLA diversity differently. Kaufman et al. (Kaufman, 2018; Di et al., 2021) have proposed promiscuous and generalist HLA categories when Di et al. have challenged the concepts of supertypes and function peptide-binding groove groups.

### HLA Data Formats

The modern and legacy nomenclature systems are still in use, which often makes data comparison and meta-analysis difficult. In addition, *HLA* alleles are stored in multiple formats which impact their use with bioinformatic tools. TSV or CSVs have been used to store HLA genotypes, usually organizing individuals in rows and *HLA* genes in columns (with two columns for each gene). Such files are often generated manually, but are used by multiple population genetic and disease-association applications (Lancaster et al., 2007; Excoffier and Lischer, 2010; Pappas et al., 2016). More strictly-defined bioinformatic-oriented formats include HLA PED (or HPED) (Choi et al., 2021), an HLA-focused extension of the PED format (Purcell et al., 2007); Variant Call Format (VCF), as used by BEAGLE (Browning et al., 2018), in which HLA allele names are recoded as multiple binary identifiers, and Histoimmunogenetic Markup Language (HML), an XML format developed specifically for exchanging HLA and Killer-cell Immunoglobulin-like Receptor (KIR) genotype data (Milius et al., 2015).

The IPD-IMGT/HLA Database releases new and updated reference sequences and allele names every 3 months. Individuals datasets may have been generated under any release version, which is why tools like the Allele Name Translation Tool (ANTT) have been developed to standardize datasets to a common release version. (Mack and Hollenbach, 2010). Development of a standardized means of storing and sharing data is still underway. In 2015, the MIRING reporting guideline (Mack et al., 2015) introduced standardized data elements and a controlled vocabulary for HLA genotype data and meta-data, which were implemented in HML (Milius et al., 2015). An HML message includes information on the IPD-IMGT/HLA Database version, the entity and how they generated the data, as well as references to external sources (e.g., reference sequences and aligned read). HML is used to transmit HLA genotyping data to the National Marrow Donor Program (and other similar registries and donor centers), but has yet to be adopted for genetic–analysis applications. Most of the existent HLA analysis applications require fewer data elements than are included in an HML message.

Given the number of different applications of HLA data, new informatics tools can influence the interpretation of this information. Multiple ancillary tools have been developed for HLA research. Whether they allow researchers to run rapid association analyses, extract new information from data, or link HLA genotypes to novel fields of translational research, all contribute to the advances in the HLA research (Table 1).

**TABLE 1:** Tools for HLA analyses.

HLA application name	Description	URL
Alphlard-nt (Hayashi et al., 2019)	Identification of somatic mutations in HLA molecules from whole-genome and exome data using Bayesian algorithms	—
BIGDAWG (Pappas et al., 2016)	Open-source R package for the case-control analysis of highly polymorphic data at the allele, haplotype and amino-acid level	https://CRAN.R-project.org/package=BIGDAWG
Easy-HLA (Geffard et al., 2020)	Website with HLA alleles haplotyping, upgrading and inference from HLA genotypes, prediction of HLA-C expression	http://hla.univ-nantes.fr/
HATK (Choi et al., 2021)	Open-source *Python* pipeline for HLA association studies, including tools for HLA data formatting	https://github.com/WansonChoi/HATK
HLA-check (Jeanmougin et al., 2017)	Perl tool evaluating the probability of accurate HLA genotype imputation by comparing it to SNP imputation in the exonic region of HLA.	https://github.com/mclegrand/HLA-check/
HLA-EMMA (Kramer et al., 2020)	Donor/recipient compatibility assessment based on solvent-accesible amino acids, based on intralocus comparisons	http://www.HLA-EMMA.com
HLA*fix*	Open-source R pipeline for HLA association studies. Performing SNP quality control steps, stratification, HLA imputation and representation of the results	https://univ-nantes.io/Nico_V/hlafix
HLAHapV (Osoegawa et al., 2016)	A Java-based HLA Haplotype Validator for quality assessments of HLA typing	https://github.com/nmdp-bioinformatics/ImmunogeneticDataTools
HLA-NET (Nunes et al., 2014)	Set of tools to manipulate HLA data, infer haplotypes, convert files format, and information about typing	https://hla-net.eu/
HLApers (Aguiar et al., 2020)	Genotyping and quantification of HLA expression from RNA-seq data	https://github.com/genevol-usp/HLApers
HLA-TAPAS (Luo et al., 2020)	Open-source *Python* pipeline for creation of reference panels and HLA association studies	https://github.com/immunogenomics/HLA-TAPAS
MergeReference (Cook and Han, 2017)	SNP2HLA compatible tool to concatenate multiple reference panels in order to gain accuracy during HLA imputation	http://software.buhmhan.com/MergeReference
pyHLA (Fan and Song, 2017)	Association analysis for HLA alleles in *Python* language	https://github.com/felixfan/PyHLA

## Inferring and Imputing *HLA* Alleles: From Complex Read-Mapping to the Study of Linkage Disequilibrium

*HLA* inference is an umbrella term comprising multiple bioinformatic tools and statistical methods to obtain individuals’ *HLA* genotypes. Inference implies using missing information to obtain *HLA* genotypes, this can generally refer to using untargeted sequencing data, which have insufficient sequence read depth, to thoroughly recover the *HLA* alleles polymorphisms (Klasberg et al., 2019).

### Inference From Whole-Genome Sequencing and Whole-Exome Sequencing

Unlike NGS typing techniques which targets *HLA* genes (as many commercial kits apply), untargeted sequencing does not focus on *HLA*. Whole-genome sequencing (WGS) methods aim to identify all genetic variations of an individual genome, while whole-exome sequencing (WES) is designed to target all exons. Initially, these methods did not support the calling of *HLA* alleles; low coverage and short read-lengths led to poor *HLA* typing accuracy (Bauer et al., 2016). Low coverage does allow identification of *HLA* alleles, due to their high levels of polymorphism and extensive conserved sequences among genes, and improvements were needed (Hosomichi et al., 2015). Moreover, general pipelines for analyzing NGS data from WGS do not work for *HLA* genes; because they present high sequence similarity, it is very common that a short read (a sequence generated in NGS procedures) from one gene aligns to another gene (cross-mapping), leading to genotyping errors (e.g., *HLA-A* and *HLA-H*, or *HLA-C* and *HLA-B*) (Castelli et al., 2018). The intense polymorphism observed in *HLA* genes may bias read alignment when using a single genome reference, especially when one individual presents too many modifications compared to the reference genome. This issue overestimates reference allele frequencies and causes genotyping errors (Brandt et al., 2015). Therefore, it is mandatory to use methods tailored for *HLA* genes to get reliable genotypes and haplotypes at the SNP level from NGS data.

Multiple algorithms have been developed and refined (Klasberg et al., 2019). These include: 1) classic read-mapping with HLA-specific quality control steps or different scores, hla-mapper (http://www.castelli-lab.net/apps/hla-mapper) (Castelli et al., 2018) which also works on KIR genes and provide genotyping and haplotyping at the SNP level, seq2HLA (Boegel et al., 2012) and HLAforest (Kim and Pourmand, 2013), among other tools; 2) population graph reference methods (e.g., HLA*PRG:LA), which identify probability edges between polymorphisms nodes and project read data onto these to evaluate the most likely alleles.

Recent reviews and tool comparisons on the optimal methods for non-*HLA* targeted sequencing data are already available (see (Klasberg et al., 2019; Chen et al., 2021)). In 2020, Chen et al. found that HLA-HD was the most accurate tool for producing HLA genotypes from WGS and WES. However, the study focused on the performance of five tools only. Notably, most of the tools they studied achieved much higher accuracies than previously reported by Bauer et al., 2016, which emphasizes a drastic improvement in read coverage and processing in the *MHC* region (Bauer et al., 2016). Finally, researchers successfully implemented these tools in association studies, promoting their importance for HLA-centric epidemiological studies (Juhos et al., 2015; Xie et al., 2017; Mimori et al., 2019; Vince et al., 2020a).

### *HLA* Allele Imputation

*HLA* genotyping data can also be generated using *HLA* imputation tools, which generate genotypes for individuals on the basis of LD between GWAS-derived SNP data for the *MHC* region and specific *HLA* alleles. These methods ultimately rely on reference datasets of *HLA* and SNP genotypes for the same individuals, and have become increasingly accurate in their predictions as new algorithms are developed.

**FIGURE 2:**
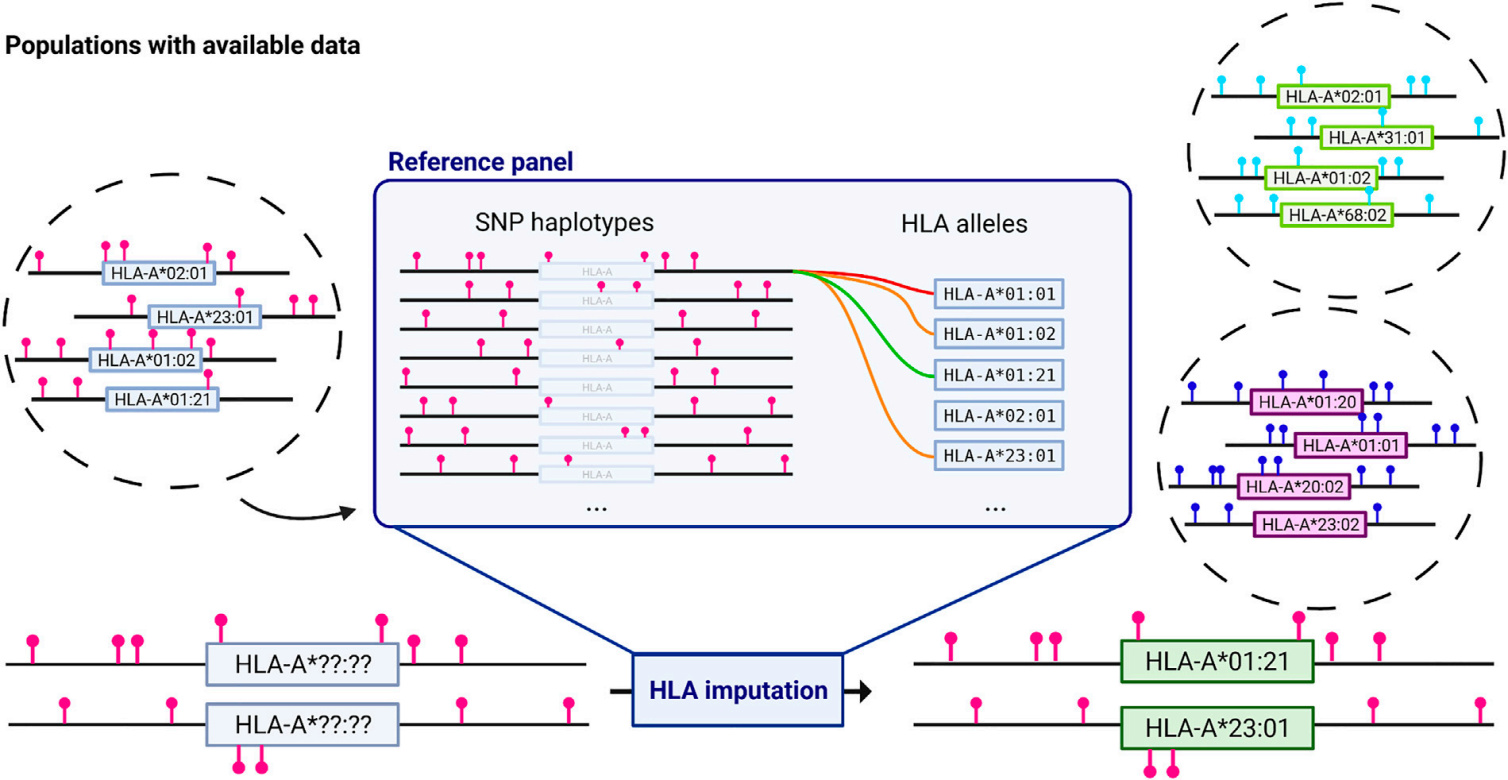
*HLA* imputation from GWAS data. Reference panels are created from individuals with known SNP and *HLA* data. Depending on the method, an algorithm will deduce the probability of a specific *HLA* allele in the population given a SNP haplotype. These new found links are stored for that reference panel and applied to new SNP data to infer *HLA* genotypes. *HLA-A* is given as an example with a truncated list of alleles; other *MHC* genes are imputed using the same method. Different populations are represented in different circles and imply different allele frequencies. Pinpoints represent SNPs and are only indicative. *HLA* imputation results are highly dependent on the population chosen for the reference panel. Created with biorender.com.

Following the opportunity brought by SNP to SNP imputation, SNP to *HLA* imputation algorithms offered a quick and easy way to obtain *HLA* genotypes from widely available GWAS SNP genotyping data (McCarthy et al., 2016). SNP to *HLA* imputation relies on reference panels of individuals with known SNPs and *HLA* genotypes, to generate links between SNPs, haplotypes, and *HLA* alleles using machine learning algorithms (Figure 2).

The first published algorithms, SNP2HLA (Jia et al., 2013) and HLA*IMP (Dilthey et al., 2011), were based on different implementations of hidden Markov models; SNP2HLA used BEAGLE (Browning and Browning, 2009), a haplotyping and SNP genotype imputation tool. In 2014, Zheng et al. proposed HIBAG, an attribute bagging method tailored for *HLA* data (Zheng et al., 2014), which showed better performance than pre-existing tools, and at the time was the only method to provide population-specific reference panels for hundreds of individuals while enabling construction of personalized reference panels building. Initial independent reviews suggested that SNP2HLA performed better on 3,265 samples from BioVU, a de-identified electronic health record database coupled to a DNA biorepository (Karnes et al., 2017). However, later reviews (Kuniholm et al., 2016; Pappas et al., 2018) and studies (Ritari et al., 2020) have favored HIBAG for HLA imputation, notably on more complex HLA data.

In practice, both SNP2HLA and HIBAG are commonly used to conduct *HLA* imputation or creation of new reference panels. Overall accuracy differences are low for European panels that had been extensively assessed. An important point still under investigation is the impact of population diversity in reference panels. While some researchers advocate for the creation of exhaustive multi-ethnic reference panels (Degenhardt et al., 2019), others have shown that specific populations (e.g., insular or admixed require more restrained reference panels (Khor et al., 2015; Ritari et al., 2020).

The difficulty in determining if a reference panel is suitable for *HLA* imputation is related to how well it matches to target data, on the frequency of common alleles and the presence of rare *HLA* alleles, specific to some population (especially in underrepresented populations). This has led to the creation of reference panels with limited *HLA* diversity. While accuracy values are often reported as the ultimate answer to a model viability, these values can be misleading. For a rare *HLA* allele in a validation dataset, a 90% accuracy value can be achieved if that allele should be imputed 20 times out of 2,000 alleles (i.e., 1,000 individuals) but is never predicted. Therefore, other metrics (e.g., sensitivity, specificity, or F1 score (Cook et al., 2021)), must not be overlooked. Admixed populations are formed by individuals from different genetic backgrounds in variable proportions, and *HLA* imputation can be sub-optimal if the reference panel is only drawn from one of the ancestral populations. Conversely, a reference panel from an admixed population with a different overall genome proportion from the individuals being imputed may also provide inaccurate results.

To effect worldwide improvement in *HLA* imputation efforts, we led the creation of an international consortium, the SNP-HLA Reference Consortium (SHLARC), whose aim is to gather data to represent the extreme diversity of *HLA* alleles, fostering accurate imputation (Vince et al., 2020b). We further advocate for improvements to current *HLA* imputation tools and for the development of a platform promoting easy access to *HLA* imputation for immunogeneticists. Though *HLA* imputation is not yet suited for clinical settings, generalization of *HLA* association studies offers a new way to investigate immune pathologies (Meyer and Nunes, 2017).

New versions HLA*IMP (Motyer et al., 2016) and SNP2HLA have been released (e.g., MHC*IMP (Squire et al., 2020), CookHLA (Cook et al., 2021), and Deep-HLA (Naito et al., 2021)) that apply new algorithms. These highlight the community intense interest in *HLA* imputation. CookHLA is an updated version of SNP2HLA (based on the BEAGLE algorithm) that better accounts for LD in the *HLA* region and makes use of the genetic map option to better impute individuals who are not well represented in the reference panels. For its part, Deep-HLA seems especially promising as deep learning may lead to better imputation of rare alleles.

## Bioinformatic Analyses of HLA Information

The pressing challenge of understanding the COVID-19 pandemic, given previous associations with infectious diseases, has led researchers to scrutinize *HLA* using any available resource. In addition to issues of nomenclature and on-going technological evolution of typing methods, the complexity of *HLA* analyses is also derived from the multiple forms these analyses can take. On the one hand, *HLA* allele frequencies and predicted binding affinity of pathogen peptides to *HLA* alleles allow for a first step in the HLA world, as they are easily available, but are limited to investigate its actual role. On the other hand, the in-depth implication of *HLA* is revealed when looking at SNP association in the MHC region, and specifically when looking at allele associations, but their realization is hindered by high costs and technical difficulties. The study of HLA is multi-layered, with a continuum of methods peaking with analysis of individual data and multi-locus haplotypes, all of which contributing to a comprehensive understanding of the role of HLA in a given analysis.

### *HLA* Allele Frequencies

The diversity of *HLA* alleles across geographically separated populations is thought to be the result of balancing selection due to local pathogens (Meyer and Thomson, 2001). The allelefrequencies.net database has the most extensive collection of *HLA* allele frequencies in diverse populations (Middleton et al., 2003). In addition, *HLA* typing conducted by bone marrow registries may constitute a local estimation of *HLA* allele distribution in a population (Sacchi et al., 2019; Schmidt et al., 2020). It is possible to statistically analyze the correlation (e.g., *via* linear regression or Pearson coefficient) between a quantitative value, such as the number of COVID-19 cases, and the *HLA* allele frequencies obtained from a different sample in every studied population (e.g., in a database or registry).

However, while these correlations are faster and easier to obtain than new *HLA* genotypes, they may result in spurious correlations because: 1) most of the *HLA* alleles (and observed haplotypes) have a low frequency. For example, according to allelefrequencies.net, in the 416,581 individuals from the African-American NMDP population in the United States, two-thirds of the 321 *HLA-B* alleles at two-field resolution have frequencies below 0.003% (24 or less occurrences). Assuming that reference population samples are representative is not always accurate. A possible solution is to focus on common *HLA* alleles; 2) statistical tests are often applied without multiple-testing correction, regardless of the number of tests; 3) the confounding variables, both genetic (e.g., ancestry) and environmental (e.g., comorbidities), are often overlooked.

In any case, *correlation* is not *causation.* Therefore, the high number of *HLA* alleles and biased frequencies are bound to create spurious links between their presence and any phenotype. Therefore, to thoroughly investigate the relationship between *HLA* and phenotype, it is of the utmost importance to conduct studies and control for other genetic factors such as population stratification, linkage disequilibrium, or comorbidities (some linked to *HLA* polymorphism itself such as diabetes). Statistical bias could also be reduced by working on a higher number of samples and correcting for multiple testing. It is also worth considering different resolution levels of information, from *in silico* studies to full haplotype information.

### *In silico* Peptide Binding

HLA molecules present endogenous and exogenous peptides, however, affinities for these peptides vary greatly depending on the peptide conformation and the peptide cleft topology and chemistry. Whether an *HLA* allele presents several or few peptides derived from one specific pathogen is one mechanism potentially explaining the strong immune response or tolerance towards it. Researchers can use prediction tools, such as NetMHCpan (Nielsen and Andreatta, 2016; Jurtz et al., 2017), trained on binding affinity and elution assays, to evaluate the number of potentially bound peptides for any *HLA* class I allele. The “pan” methods, contrary to the “allele-specific” methods, use similarities in sequence data to predict the peptide binding capacity of HLA alleles for which no information is available. Other tools exist and have been reviewed by Mei et al., in 2020 (Mei et al., 2019). Such predictions, coupled with *HLA* genotype data of individuals, give a theoretical insight into the possible adaptive immune response of a person. In these tools, the peptidome of the studied pathogen is informatically divided into peptide sequences of limited size (8–12 residues to account for the size of peptides presented by class I molecules), and the number of alleles predicted to bind a large number of peptides is inferred to represent better presentation to T cells, and a protective role against the pathogen. However, the only way to definitely determine peptide binding affinity is through laboratory experiments.

### Genome-wide Association Studies

Genotyping data obtained with SNP arrays has proven to be fast and inexpensive for investigating the genetic component of complex traits and diseases (Claussnitzer et al., 2020), compared to more thorough and exhaustive sequencing technologies. Without assumptions regarding the region potentially involved in the studied trait, GWAS helped discover protective and risk alleles, particularly in the *HLA* region (Kennedy et al., 2017). Contrary to the use of independent *HLA* allele frequencies for studying a pathology, association studies assess the difference between affected individuals and unaffected individuals or the distribution of a particular quantitative trait. Both genetic and phenotypic data are individual and not population-based, reducing biases. The statistically significant SNP (aka, top hits) are linked to genes by proximity, and investigation by pathway analysis can reveal additional biological information on their effect. More recently, transcriptome-wide association studies have allowed more accurate investigation of the impact of a SNP on the expression of genes (Wainberg et al., 2019). In addition, some SNPs can be highly correlated to an *HLA* allele (e.g., rs2395029 and HLA-B*57:01 have been described multiple times as in complete linkage disequilibrium (de Bakker et al., 2006)), and therefore provide additional functional information for biological interpretation. Finally, statistical regression models can take into account potential confounding factors (e.g., genetic ancestry and population stratification, sex, age, comorbidities) to control for limiting biases.

Given the complex LD patterns across the *MHC* region, SNP association analyses are not usually precise enough to identify specific disease-associated *HLA* alleles. LD patterns may differ between populations. For example, the rs2395029 tags HLA-B*57:01 in Europeans but displays reduced LD in African-Americans (Colombo et al., 2008). The complex LD patterns and the high number of genes in the *MHC* region, make it difficult to pinpoint an SNP to a specific *HLA* allele in most cases.

### *HLA* Allele Association Studies

Association studies of *HLA* alleles offer a more relevant biological explanation, based on peptide presentation. *HLA* allele data can come from different sources, including various epochs of *HLA* typing and *HLA* imputation from SNPs (see above). These data can be analyzed as is, or low resolution HLA data can be “upgraded” using the HLA-Upgrade tool from the Easy-HLA website, which statistically impute the most probable two-field genotype based on a haplotype database (Geffard et al., 2020). Once HLA data from multiple sources have been standardized for allele content and resolution, a frequency cut-off value is usually applied to test only those alleles with sufficient occurrences in the dataset to guarantee statistical power in the analysis. *HLA* alleles being highly polymorphic, they often display lower frequencies, and a larger sample size is usually required to obtain significant results compared to SNP analyses.

Regression models, which are commonly used for SNP association, are the most versatile and common statistical models implemented to test associations between individual *HLA* alleles and phenotypes of interest (linear models for continuous and logistic for discrete phenotypes, respectively). Regression models can work with multiple covariables, allowing the disentanglement of the HLA effect and confounding factors such as population stratification, sex, gender, and others. Similar to GWAS SNP analyses, *HLA* alleles are tested individually as biallelic markers for each *HLA* gene, as each individual can exhibit 0, 1, or 2 occurrences of a given allele. As HLA molecules are expressed co-dominantly (Hughes and Nei, 1988), the dominant genetic model is commonly preferred to allelic or recessive models to assess *HLA* allele associations. However, it should be mentioned that different alleles might present different expression levels due to promoter and 3′UTR variations and final protein stability. Indeed, this is another HLA world: the effect of variants in the expression levels, which sometimes are directly linked with disease susceptibility (Kulkarni et al., 2011).

As in GWAS analysis, the overall performance of a statistical model can be evaluated with a Quantile-Quantile (QQ) plot, representing the observed *p*-value distribution for each *HLA* allele compared to the expected distribution under the null hypothesis. Any deviation from this distribution is highlighted by a deviation from a straight line. (Murdoch et al., 2008). Different scenarios can be described: 1) observed *p*-values mostly follow the null hypothesis, indicating that the statistical model accurately fits the data; 2) observed *p*-values deviate below the null hypothesis line, indicating that the statistical model is probably underpowered; 3) observed *p*-values deviate above the null hypothesis line, indicating that the statistical model may not be well parameterized and some confounding factors are not enough considered. Once the robustness of the analysis is confirmed, it is important to obtain a comprehensive visualization of the results with Manhattan plots, for instance, displaying–log10 (*p*-value) along with the list of test *HLA* alleles ordered numerically (as seen in Vince et al. (Vince et al., 2020a)). Volcano plots can also display the significativity of alleles along with their effect size, allowing a global view of their impact. Finally, the significance threshold accounting for multiple testing can be determined with the Bonferroni correction (5% α threshold divided by the number of tests) or other corrections such as the FDR, or permutations.

### Easy-HLA: Going Beyond *HLA* Alleles to *HLA* Genes Haplotypes, HLA Expression Levels, Specific HLA Amino Acids, KIR Ligand Groups

**FIGURE 3:**
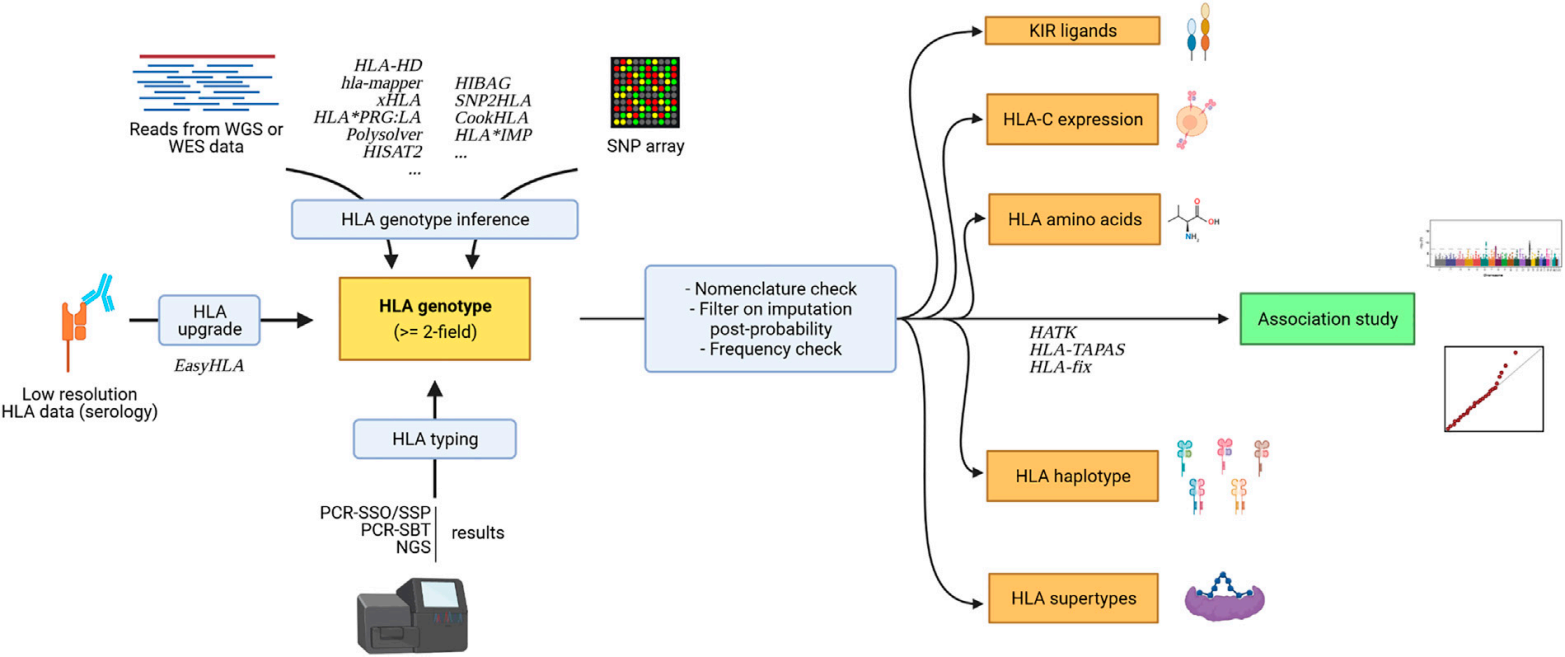
Association study pipeline for HLA data and surrounding immunogenetic factors. Created with biorender.com.

New tools have been developed to facilitate the analysis of additional immunogenetic parameters (e.g. KIR ligands, see Figure 3).

*HLA* genotypes can be used to infer additional immunogenetic parameters that can further be analyzed (see Figure 3.) to get a clearer understanding of the relationship between immunity and pathologies. While one *HLA* allele already represents a haplotype of SNPs within a gene, as it is a collection of polymorphisms in the gene of interest, researchers have demonstrated the importance of looking at multiple *HLA* alleles on the same chromosome, which is referred to as an *HLA* haplotype. Association studies can be done on haplotypes, but many haplotype frequencies can be even lower than constituent allele frequencies. In a clinical setting, the collection of haplotype information is also useful, notably in HSCT transplants, for identifying haploidentical individuals. These haplotypes can be inferred using the HLA-2-Haplo tool from Easy-HLA website (Geffard et al., 2020), for instance. A straightforward, reliable, but expansive strategy to get *HLA* gene haplotypes is the analysis of trios (mother, father, and offspring) or third-generation long-read sequencing such as PacBio SMRT.

Easy-HLA also infers HLA-C expression levels, HLA alleles amino acids, and KIR ligand groups. Recently, high HLA-C expression levels were associated with better control of HIV (Apps et al., 2013; Vince et al., 2016). Class I *HLA* alleles have also been grouped according to their dependence on tapasin, a major actor in peptide loading, which proved to be an interesting subdivision for studying HIV-1 control (Bashirova et al., 2020). Moreover, testing HLA allele amino acids may indicate a specific function of a given residue across several alleles, as with this study by McLaren et al., again in HIV control (McLaren et al., 2012). Finally, studying KIR ligand groups along with KIR typing as previously described (Martin and Carrington, 2013; Vince et al., 2014) can reveal the binding patterns of specific HLA alleles. For example, HLA-A and HLA-B molecules bearing the Bw4+ motif bind specifically to KIR3DL1. Similarly, HLA-C group 1 (C1) allele-encoded molecules carry an asparagine at position 80 and specifically bind KIR2DL2/3, as opposed to group 2 (C2) allele-encoded molecules, which carry a lysine and specifically bind KIR2DL1 (Parham et al., 2012). Grouping *HLA* alleles according to different functional parameters can increase the power of detecting a true positive signal and represent an opportunity to come closer to the biological cause behind *HLA* genetic association with diseases.

## Conclusion

However intricate it may be, the *MHC* region, and *HLA* in particular, is the perfect candidate to investigate infectious or auto-immune diseases, as its primary biological role is to present antigen to the immune system. HLA research was able to grow in different directions from *in silico* studies on peptide binding to association studies of *HLA* alleles, giving leads on HLA involvement in pathologies. That said, HLA-focused analysis requires special care because its immense diversity and low-frequency distribution may potentially result in spurious associations when tested incorrectly or in a small cohort. Fortunately, many tools have been and are still developed to obtain high-quality HLA information for a low cost with statistical inference, through *HLA* inference from NGS data or *HLA* imputation from SNP GWAS data or HLA resolution upgrading from HLA genotypes. Researchers considering to explore HLA should take advantage of existing resources and mobilize them when taking on new challenges, such as with the SARS-CoV-2 research.
